# Preparation and In Vitro Characterization of Gels Based on Bromelain, Whey and Quince Extract

**DOI:** 10.3390/gels7040191

**Published:** 2021-10-30

**Authors:** Amalia Mazilu, Violeta Popescu, Codruta Sarosi, Radu Silaghi Dumitrescu, Andrea Maria Chisnoiu, Marioara Moldovan, Laura Silaghi Dumitrescu, Doina Prodan, Rahela Carpa, Georgiana Florentina Gheorghe, Radu Marcel Chisnoiu

**Affiliations:** 1 Physics and Chemistry Department, Technical University of Cluj-Napoca, 28 Memorandumului Street, 400114 Cluj-Napoca, Romania; amalia.mazilu@gmail.com (A.M.); violeta.popescu@chem.utcluj.ro (V.P.); 2 Department of Polymer Composites, “Raluca Ripan” Institute of Research in Chemistry, Babes-Bolyai University, 30 Fantanele Street, 400294 Cluj-Napoca, Romania; liana.sarosi@ubbcluj.ro (C.S.); laura.silaghi@ubbcluj.ro (L.S.D.); doina.prodan@ubbcluj.ro (D.P.); 3 Department of Chemistry, Faculty of Chemistry and Chemical Engineering, 11 Arany Janos Street, 400028 Cluj-Napoca, Romania; rsilaghi@chem.ubbcluj.ro; 4 Department of Prosthodontics, “Iuliu Hatieganu” University of Medicine and Pharmacy, 32 Clinicilor Street, 400006 Cluj-Napoca, Romania; 5 Department of Molecular Biology and Biotechnology, Faculty of Biology and Geology, Babeș Bolyai University, 1 M. Kogălniceanu Street, 400084 Cluj-Napoca, Romania; rahela.carpa@ubbcluj.ro; 6 Faculty of Dental Medicine, Carol Davila University of Medicine and Pharmacy, 17-23 Calea Plevnei, 010232 Bucharest, Romania; georgiana.gheorghe@umfcd.ro; 7 Department of Odontology, Endodontics and Oral Pathology, “Iuliu Hatieganu” University of Medicine and Pharmacy, 33 Motilor Street, 400001 Cluj-Napoca, Romania; marcel.chisnoiu@umfcluj.ro

**Keywords:** bromelain, whey, whitening gel

## Abstract

The growing interest in the appearance and color of teeth has led to the emergence of a wide range of teeth whitening methods, both in dental offices and in patients’ homes. Concerns about the possible side effects or toxic effects of peroxide-based whitening gels leads to the identification of alternative whitening methods, based on natural compounds with mild action on tooth enamel and remineralizing effect. In this context, this study describes the preparation and in vitro analysis of whitening gels based on natural active agents—bromelain, quince and whey—using organic (polyacrylate, polyethylene glycol) and/or inorganic (silicate) excipients. Five natural products gels were prepared, containing bromelain extract, quince extract and whey, in various proportions. Two supplementary gels, one containing Lubrizol and another containing SiO_2_, were prepared. All gels were submitted for multiple in vitro analysis such as: SDS-PAGE analysis, UV-vis and FTIR spectroscopy, SEM microscopy, antibacterial activity on Streptococcus mutans ATCC 25175, Porphyromonas gingivalis ATCC 33277, Enterococcus faecalis ATCC 29212, *Escherichia coli* ATCC 25922 and Staphylococcus aureus ATCC 25923. The quince extract sample was the only one which completely discolored the blue dye on SDS-PAGE analysis. On the UV-vis spectra, the 303 nm band is assigned to an in situ modified form of bromelain. SEM images of gels containing SiO_2_ particles show evident marks of these particles, while the rest of the gels containing Lubrizol or whey are more uniform. Regarding antibacterial tests, the SiO_2_ gel samples did not show inhibition in any strains, but the other tested samples varied in the size of the inhibition diameter depending on the amicrobial strain tested; the protease activity of bromelain modulates the composition of the added whey proteins. Bromelain added as a nanoencapsulated assembly better preserves its integrity. The prepared gels showed antibacterial properties.

## 1. Introduction

The appearance and color of the teeth are a concern for a growing number of people seeking alternatives to chemical-based dental treatment. Recently, there has been a significant development of teeth whitening products “without a prescription” and without the involvement of professionals [1]. Dental staining can result from the accumulation of molecules with chromophore/chromogenic groups (generally conjugated or even aromatic unsaturated systems) on the surface of dental structures [2]. The adhesion of chromophores to teeth is due to several types of forces, generally supramolecular—especially van der Waals. Factors related to eating habits, such as excessive coffee or tea consumption, smoking, and exposure to chemicals, are associated with dental staining. Teeth whitening products are traditionally based on hydrogen peroxide or carbamide peroxide gels (hydrogen peroxide and urea) [3]. These peroxide-based products release free radicals that attack chromogens, being able to degrade larger molecules into smaller molecules that can be easily removed from dental tissues, thus promoting a certain degree of teeth whitening. Despite their effectiveness, peroxide-based products can cause severe abrasion of dental structures and are toxic/corrosive to any tissue in general. However, new studies highlighted that a low percentage of hydrogen peroxide, together with new nanofillers, allows for maintenance of enamel structure [4]. In this context, attempts are being made to develop whitening products with enzymatic action, which in some cases seem to be just as effective in removing extrinsic stains. Active ingredients derived from natural products have been shown to have potential antibacterial effects without causing abrasiveness, which can be a good alternative to those based on peroxides [5].

Bromelain is an enzyme extract with protease activity, which is found mainly in the pineapple plant (Ananas comosus) of the genus Bromeliaceae [6]. This extract can be obtained from both the stem and the fruit of the pineapple plant and contains as the main component a mixture of glycosylated proteolytic sulfhydryl enzymes [7,8,9,10,11]. The bromelain strain possesses different biochemical properties and compositions compared to fruit bromelain [12], the latter containing several thiol endopeptidases and also compounds such as peroxidases, acid phosphatase, glycoproteins, carbohydrates and organic complexed Ca^2+^ [6,13]. To date, eight active proteolytic components have been isolated from bromelain [14]. Proteinases are considered to be the most active fraction, comprising ~2% of total proteins; occasionally, the term bromelain is also used to describe only the two dominant proteases in this extract [15]. Bromelain works in a pH range of 4.5 to 9.5 [16]. Bromelain is one of the most widely investigated proteolytic enzymes/extracts for practical and industrial applications, due to its ease of extraction and low cost given by the relatively affordable raw material. Because of its protease activity, bromelain has been cited for its anti-edematous, fibrinolytic, anticancer, anti-inflammatory, antimicrobial, anticoagulant, and antithrombotic medical applications—generally due to the ability of bromelain to degrade connective proteins from inflamed tissues or tissues, or circulating proteins in the blood (such as those involved in coagulation), or proteins in pathogens (leading to the death of those agents—hence antimicrobial activity) [17].

In addition to the clinical approach, bromelain has been used in various industries, including the food industry [13], such as breweries [18], meat processing and tenderizing, textile and cosmetics [19]. An advantage for some industrial applications is that the optimum temperature of bromelain is ∼50–70 °C [20]. 

Toothpastes based on proteolytic enzymes have proven their effectiveness by removing tooth stains, this being done with reduced roughness [5]. Indeed, for dental applications—such as toothpaste or whitening solutions—the main candidates as alternatives to peroxides are cysteine-protease enzymes, such as papain and bromelain, described as active agents with whitening potential [5,20,21,22,23]. Proteases disrupt or remove the portion of protein in the film layer that forms on the surface of the teeth, thus removing the pigments that are bound to them. Muchow et al. [21] conducted a study on the whitening effect of whitening gels containing proteolytic enzymes (bromelain or papain) on bovine enamel. The enamel was stained in coffee solution for 1 week and measured spectrophotometrically for color evaluation before and after whitening using a proteolytic enzymes-based gel and a commercial bleaching gel with 20% carbamide peroxide for comparison. The materials were applied once a week, three times a day, for 4 weeks. Bromelain and papain gels were effective in discoloring stained tooth enamel, even though their effectiveness was less than that of the carbamide peroxide product [21].

Whey, as a by-product of milk processing, contains a mixture of α-lactalbumin (15–20%), β-lactoglobulin (55–60%), and other proteins such as bovine serum albumin, lactoperoxidase, imogloblobulin, lactoferrin, phospholipoproteins, as well as enzymes, constituting a natural reservoir of bioactive peptides with physiological and antimicrobial properties, the release of which requires the hydrolysis of precursor molecules by digestive proteases or by fermentation with proteolytic microorganisms [24,25]. Whey proteins have been proposed as possible ingredients in teeth whitening products.

Another possible source of bleaching agents is plant extracts. Organic acids, such as malic acid, can promote the degradation of bacterial plaque and therefore help to remove colored compounds from them [26]. Herbal based products for dental hygiene have been also proved to have anti-inflammatory properties [27].

The present study reports the preparation and characterization of gels based on natural products—bromelain extract, quince extract, whey with potential applications in oral microbiome control for gingival or oral treatments and/or tooth whitening.

## 2. Results and Discussion

### 2.1. SDS-PAGE Analysis

**Figure 1:**
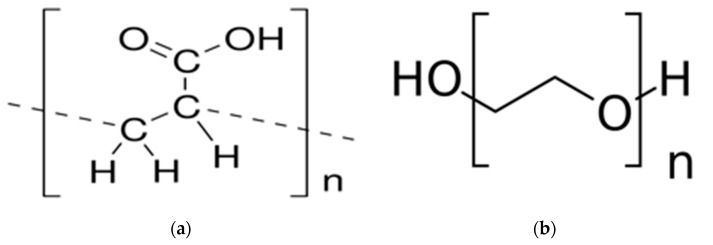
Formula of polyacrylic acid (**a**) and polyethylene glycol (**b**).

Figure 1 shows SDS-PAGE data on the gels as an analysis specific to the protein material, as most of the analyzed gels contain such material (bromelain and/or whey). Thus, all samples G1–G7 were analyzed, except the G6 gel with SiO_2_ which does not have organic matter. Bromelain is detectable both in the control sample and in the gels in which it was added as such (G1–G3) or added in the form of nanocapsules (G4), with a band of about 24.5 kDa according to the calculated molecular weight, but also with a less intense one at about 10 kDa. The G5 gel, which does not contain bromelain (and no whey, so no protein), shows no detectable SDS-PAGE signal. It can be noted that the most intense bromelain band is in the G1 gel. Interestingly, although the G3 gel contains more bromelain than the others, here the enzyme signal is weaker than in the G1 gel. This result can be interpreted as being due to the partial degradation of the enzyme in reaction with the whey present in sample G3 in the largest amount of the five gels.

The whey reference sample clearly contains two intense bands at about 20 kDa and 15 kDa, respectively. The band at ~20 kDa is completely absent in the G2–G4 gels, which contain whey in two different concentrations. In gels G3 and G4, the whey band from ~15 kDa is also very weak, while in gel G2 it is intensified. These data can be interpreted as suggesting the partial (but not total) degradation of whey proteins in those gels where bromelain is also present, generating smaller peptide chains, the whitening potential of which is expected to be higher than that of intact whey.

**Figure 2:**
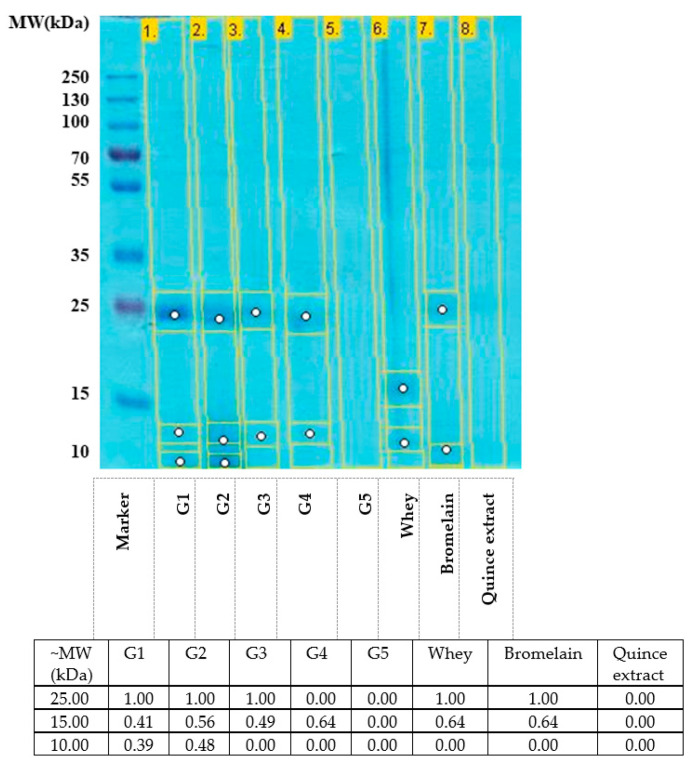
SDS-PAGE analysis of experimental gels, together with control samples (Whey, Br = bromelain, Gut. = Quince extract; each control sample was diluted to the concentrations present in the gels—with variant 0.2 for buffalo whey) and known molecular weight references. The intensities of the signals marked on the gel are calculated with the Gel analyzer program (GelAnalyzer 19.1, www.gelanalyzer.com by Istvan Lazar Jr., PhD and Istvan Lazar Sr., PhD, CSc).

The quince extract sample did not show detectable protein material in SDS-PAGE; interestingly, this sample completely discolored the blue dye (Coomassie Blue) used in preparing the sample for SDS-PAGE, which can be interpreted as further evidence of the excellent potential as a material for discoloration in the experimental gels in the present study (Figure 2).

### 2.2. UV-Vis Spectra

**Figure 3:**
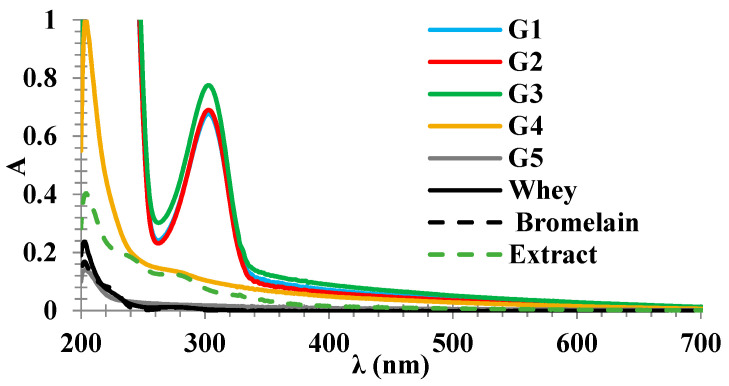
UV-vis spectra of gels and key materials in compositions.

Figure 3 shows the UV-vis spectra of gels and key materials in solution. There is an intense UV absorbance in G1–G3 gels, which is very similar between the three samples, which can be explained by the presence of polymers (silicate and polyethylene glycol in gels G1 and G2, polyacrylate, in G3) [28].

Notably, the maximum of 303 nm can be explained by neither of the polymers cited (whose absorbance is well below 300 nm), nor by the protein material: it is observed that the spectrum of bromelain and the spectrum of whey, from control samples prepared at equivalent concentrations those in whey, are much less intense than the spectra of G1–G3 and, importantly, have the maximum typical of protein material at 280 nm, not at 303 nm. However, given that G1–G3 gels have almost identical UV-vis spectra, it should be noted that their only common ingredient is bromelain. Lubrizol gel (G5), which does not contain bromelain, has negligible absorbance (and essentially represents the spectrum of the ingredient Lubrizol, based on polyacrylate and peroxide). Interestingly, the G4 gel, which has bromelain captive in nanocapsules, has an intermediate spectrum between that of G5 gel (without bromelain) and that of G1–G3 gels (with free bromelain). Based on these observations, the 303 nm band is assigned to an in situ modified form of bromelain.

### 2.3. IR Spectra

**Figure 4:**
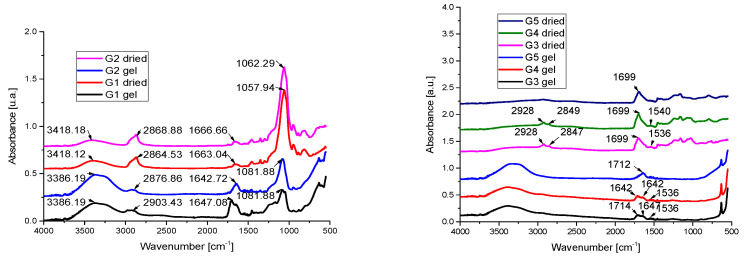
FT-IR spectra of experimental gel samples G1–G4 (according to Table 1).

Figure 4 shows the FT-IR spectra of polyethylene glycol, aerosil, and bromelain gel samples. Wet aerosil-based gel samples show intense absorption maxima around 3711 and 3032 cm^−1^ and 1645 cm^−1^. In wet samples, the absorption maxima due to the vibrations of the OH bonds in the water around 3386 cm^−1^ and 1647 cm^−1^ are especially noticeable; these maxima decrease in intensity following the evaporation of water, increasing the intensity of the maxima corresponding to the absorption bands due to the −CH_2_ group from 2928 cm^−1^. The broad band remaining after evaporation of water at large wave numbers (3711 and 303 cm^−1^) is due to the contributions of several components of the gel, so they can be attributed to the vibrations of Si-O bonds in aerosil, OH vibrations in polyethylene glycol and vibration amid A form whey. The maximum of this band underwent a shift to higher wave numbers after drying.

Dehydrated aerosil has only two absorption peaks at 1630 cm^−1^ and 3700 cm^−1^, but one may also note the presence of a wide intense band due to -OH vibrations from aerosils in the range of 3300–3700 cm^−1^, SiO-H at 3409 cm^−1^, Si-O-Si at 1130, a band that appears to be displaced both in the spectrum of wet samples and in the case of dry ones, and with a higher intensity. The characteristic bands of PEG depend on the degree of polymerization, and can be located at 3441 cm^−1^ in the case of OH stretching vibrations, 2878 cm^−1^ for CH elongation, 1464 cm^−1^ and 1343 cm^−1^ for CH deformation and 1094 cm^−1^ OH and COH.

For gels G5 and G6, the IR spectra feature the characteristic peaks of water and possibly of the amide groups, because the vibrations of the bands amide A and amide 1 are located in the same absorption range. Thus, in their case an intense band is observed with a maximum around 3700–3000 cm^−1^ due to the vibrations of the OH group in water, compared to 3404 cm^−1^, a value that corresponds to the absorption band of pure liquid water, due to symmetric stretching vibrations (v1), asymmetric stretching (v3) and bending deformation (v2) of OH, for all analyzed samples. An asymmetric absorption band can be observed in the spectra of wet gels around 1642 cm^−1^. This band can be attributed to the deformation vibrations (υ2) of the O-H bonds in the water, which can be located at 1643 cm^−1^ in the case of pure water at 25 °C. This band overlaps with the Amide I absorption band, due to vibrations of C=O groups and hydrogen bonds coupled with COO- from whey and bromelain and vibrations C=O from polyacrylic acid.

### 2.4. SEM Microscopy

**Figure 5:**
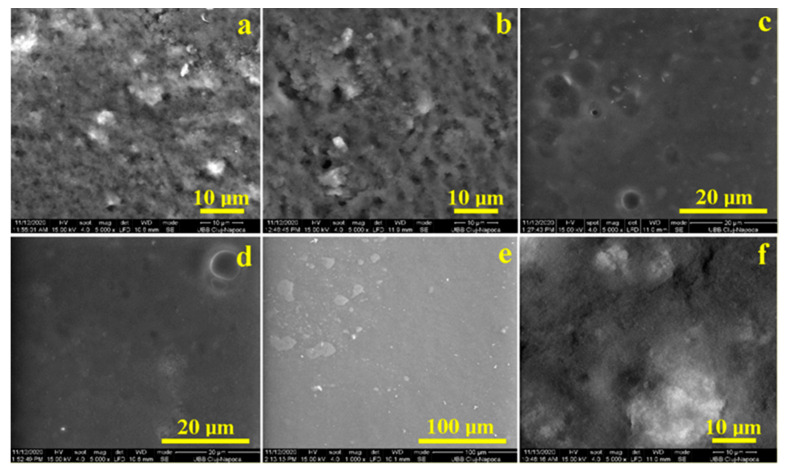
SEM images for (left to right): (**a**) G1; (**b**) G2; (**c**) G3; (**d**) G4; (**e**) G5; (**f**) G6.

In Figure 5, SEM images of samples G1, G2 and G6 and the incorporated aerosil particles may be observed, while the rest of the gels containing Lubrizol or whey are much more uniform. Gels that do not contain aerosil, but that contain the product with the enzymatic action of bromelain (3 and 4), are expected to be much more effective. These, in addition to a satisfactory whitening, are expected to produce a reduced abrasion of the surface compared to the rest of the gels, leading to fewer morphological changes on the enamel surface.

### 2.5. Antimicrobial Testing of Bleaching Gels

After the end of the incubation period at 37 °C, the zones of inhibition (mm) in the tested microbial strains were determined. It was observed that the gel sample with SiO_2_ (G6) did not show inhibition in all strains, but the other samples tested varied in the size of the inhibition diameter depending on the amicrobial strain tested (Table 1).

**Figure 6:**
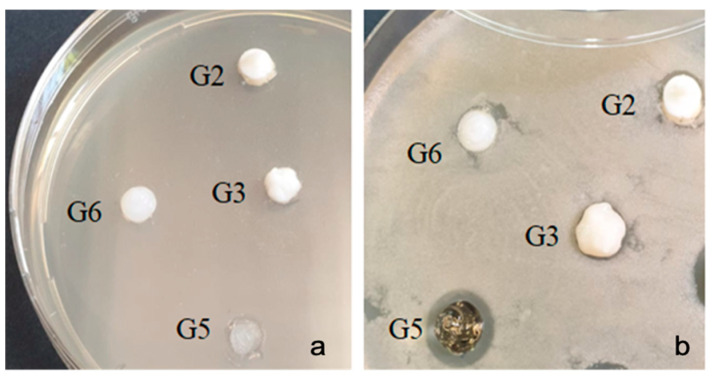
Streptococcus mutans ATCC 25175 (**a**) zero moment (**b**) inhibition after the incubation period.

With the bacterial strain Streptococcus mutans ATCC 25175, high inhibition was observed in the G5 sample (inhibition = 11 mm). Samples G2 and G3 (bromelain based gel) did not show any inhibition (Figure 6).

**Figure 7:**
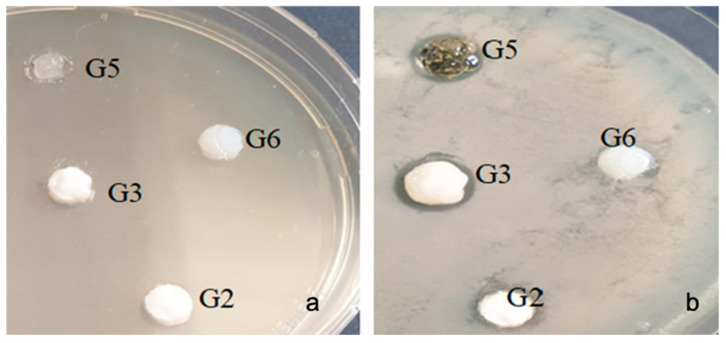
Porphyromonas gingivalis ATCC 33277 (**a**) zero moment (**b**) inhibition after the incubation period.

With the bacterial strain Porphyromonas gingivalis ATCC 33277, inhibition was observed at (9–13 mm) in all samples tested, with differences depending on the type of sample. The lowest value of inhibition was recorded in the G2 sample (bromelain based gel) (9 mm) (Figure 7).

**Figure 8:**
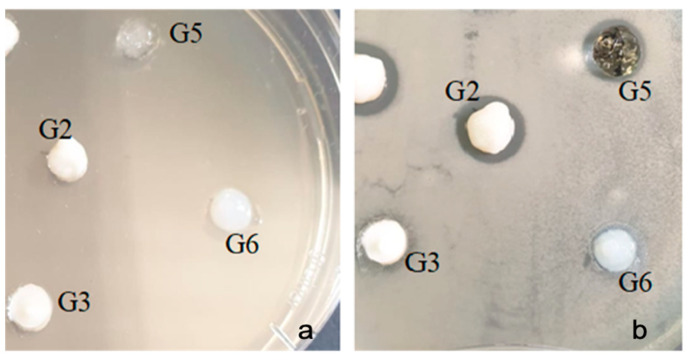
Enterococcus faecalis ATCC 29212 (**a**) zero moment (**b**) inhibition after the incubation period.

With the bacterial strain Enterococcus Faecalis ATCC 29212, inhibition was observed in all samples tested except G6. There was also a slight difference between the types of samples (8–13 mm). Sample G3 showed a slightly higher inhibition compared to the other samples (Figure 8).

**Figure 9:**
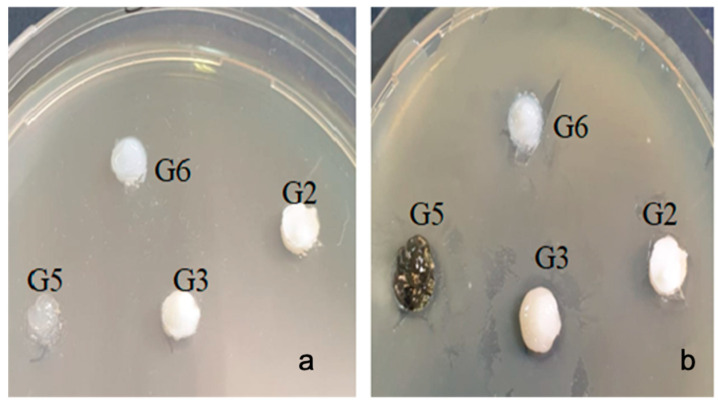
Escherichia coli ATCC 25922 (**a**) zero moment (**b**) inhibition after the incubation period.

With the Escherichia coli strain ATCC 25922, a rather low inhibition was observed and only in the G3 samples (9 mm) (Figure 9).

**Figure 10:**
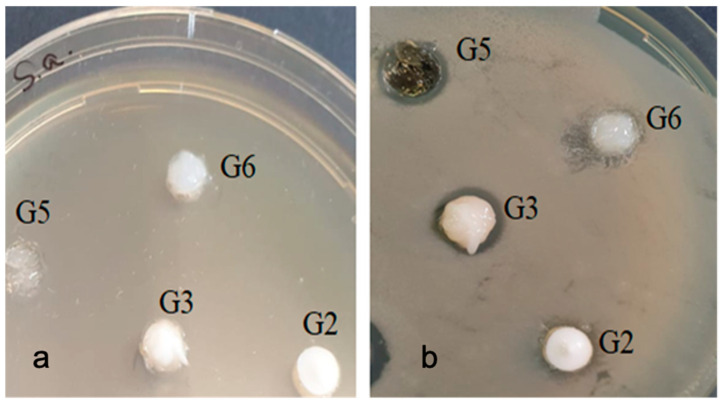
Staphylococcus aureus ATCC 25923 (**a**) zero moment (**b**) inhibition after the incubation period.

With the bacterial strain Staphylococcus aureus ATCC 25923, an inhibition was observed in all three gels, and in this case the Gel 3 sample showed a slightly higher inhibition compared to the other samples (Figure 10).

Based on the analyses reported here, one can emphasize the fact that UV-vis data in the solution can be interpreted as evidence of a transformation undergone by bromelain in G1–G4 gels. Such a transformation leaves the protein intact and active (as seen in the following sections from the fact that it is detectable in SDS-PAGE and, moreover, that the SDS-PAGE also shows evidence of its action by degrading whey proteins). The only known example to date that draws a parallel with the data in Figure 3 (bromelain spectrum intensified by an order of magnitude and with the maximum shifted above 300 nm) is that of bromelain exposed to transitional metal ions under denaturing conditions [29]. The conditions mentioned (iron, aerobic, denaturant) are ideal both for the formation of complexes with a charge transfer character from metal to ligand (characterized by significantly higher absorbance than the side chains of proteinogenic amino acids) and for the oxidation in the Fenton regime of aromatic amino acids in the protein. In contrast, the mere denaturation of the protein would not explain the increase in absorbance by an order of magnitude [30].

It should also be noted that at high concentrations of polymers such as polyethylene glycol, proteins may be partially denatured; such concentrations are found in the gels analyzed in the present experiments (PEG + Aerosil in gels G1 and G2, polyacrylate in gels G3 and G4) [9]. Under these conditions, it can be interpreted that in gels G1–G4 bromelain is partially degraded and may form complexes with traces of transitional metals in the bromelain or whey preparation. Degradation, according to the UV-vis spectrum, would be lower in the G4 gel.

This interpretation is also consistent with the SDS-PAGE data in Figure 2; their whey proteins are partially degraded in gel G2 (which has free bromelain and partially degraded according to the UV-vis spectrum) but essentially invisible (so more hydrolyzed later) in the G4 gel, where bromelain is (nano) encapsulated. For gel G3, the same trend is preserved, but it is only visible in the quantitative analysis of SDS-PAGE data in Figure 2. Thus, nanoencapsulation would allow the bromelain in the gels to have a longer lifespan. This can be seen as an advantage of the ingredient, although the partial degradation of bromelain in G1–G3 gels may itself provide an advantage by reducing selectivity (at the denaturation/partial degradation of the active site), which could increase the spectrum of protease action of bromelain in gels.

Displacement of the IR spectrum absorption bands from those of pure PEG and aerosil demonstrates the existence of strong interactions between these compounds, probably due to the formation of hydrogen bonds. It seems that due to the presence of bromelain, the bands play a less important role, probably destroying the three-dimensional structure due to the appearance of stronger hydrogen bonds of C = O • • • OH, which are formed between polyethylene glycol and bromelain. By replacing the C = O • • • NH bonds in bromelain, bromelain interactions with aerosil are also possible. On the other hand, the low concentration of bromelain in 2% whey explains the lack of clear maximum absorption of it.

Moreover, the lack of vibrations due to the amide groups in the case of the sample that does not contain whey determined the appearance of a more symmetrical absorption band in the case of the gel based on polyacrylic acid. The shape of the absorption strips suggests that an important contribution to the vibrations of the wet gel strips is due to the amide groups in the whey. The bands underwent a shift from the vibrations of the OH groups in the water, from 1643 to 1647 cm^−1^, due to the interactions with the molecules of the whey proteins.

In the case of dry samples, there is a shift in the absorption characteristic peaks of the C=O groups of polyacrylic acid for all samples, suggesting that the decrease in water concentration caused significant changes in the C = O vibrations of polyacrylic acid, which become dominant. In the spectrum of samples that contain whey, there is an increase in the intensity of the bands due to the CH_2_ groups which can be attributed to the amide group B due to the asymmetric stretching vibrations of the CH_2_ group around 2928 cm^−1^, bands that are less defined in the case of sample G5, which contains only Lubrizol. At smaller wave numbers, a band around 2880 cm^−1^ can be noticed in the case of dry G3 and G4 samples the absorption band of the CH_3_ group, due to the symmetrical stretching vibrations in the protein. Because bromelain and whey are protein-based, the main absorption bands of the two compounds overlap.

As early as 1999 [30], bromelain has been shown to act as an antibacterial agent by inhibiting the growth of intestinal bacteria, such as *Escherichia coli*, by helping to stop the production of enterotoxins by these bacteria. In 2014, the antibacterial effect against strong periodontal pathogens was studied [31], showing that this enzyme also demonstrates an anthelmintic effect against gastrointestinal nematodes, such as *Heligmosomoides polygyrus*, *Trichoderma viride* and *Trichurismuris* [32]. Following antimicrobial tests on gels enriched with bromelain, it can be stated that they have different antimicrobial activity depending on the type of extract and depending on the bacterial strain tested. No inhibition was recorded in the SiO_2_ gel sample, which means that in the gel samples tested only the active compounds in them acted on the bacteria and not the solvent.

Currently, bromelain is administered for many clinical applications due to its therapeutic effects in the treatment of inflammation and soft tissue lesions. The studied gels can be used for tooth whitening gels and other gingival or oral treatments. More evidence is necessary to establish the efficiency and safety of these products for use in dentistry.

## 3. Conclusions

Beyond ensuring the desired physical properties for application as whitening gels, the excipients modulate and allow the preservation of the integrity and/or properties of natural ingredients. The protease activity of bromelain modulates the composition of the added whey proteins. Bromelain added as a nano encapsulated assembly better preserves its integrity, showing different antimicrobial activity depending on the type of extract and depending on the bacterial strain tested. Health professionals can use bromelain gel for its therapeutic effects in oral injuries of soft tissues, and they can explore its properties as a whitening tool for dental enamel.

## 4. Materials and Methods

### 4.1. Preparation of Experimental Gels

Bromelain-based gels were obtained using polyethylene glycol (PEG 400) or aerosil (SiO_2_ medical products; Sigma Aldrich) and Carbopol (Lubrizol Advanced Materials, Inc., Belgium) as thickeners. Under the trade name aerosil, amorphous nanometric SiO_2_ powders of the “pyrogenic silica”, “fumed silica” or “fume silica” type are generally available. This powder, used as a thickening agent, is obtained by pyrolysis of silicon tetrachloride at temperatures above 1500°C. The carbomers used as thickeners are high molecular weight polymers of acrylic acid. As an alternative thickening agent, polyethylene glycol was also used—a polyether type polymer with the structural formula H−(O-CH_2_-CH_2_)_n_-OH.

The lyophilized whey used at this stage is a by-product resulting from milk processing, resulting from the separation of the cheese. For a better dispersion of bromelain, it was dissolved in water prior to adding the gels.

The chemical composition of the developed gels is shown in Table 2.

### 4.2. SDS-PAGE Analysis

Gel electrophoresis under denaturing conditions (SDS-PAGE) was performed for the experimental gels in order to examine the stability of whey proteins and bromelain, following an electrophoresis and quantitative analysis protocol previously described and applied to milk and whey [21].

### 4.3. UV-Vis Spectra

Alternatively, the gels were dissolved in distilled water and measured in solution using a Cary 50 UV-vis apparatus (Varian, Inc., Foster City, CA, USA), and Lambda 25 (PerkinElmer Singapore) spectrophotometers. The wavelengths ranged from 200 to 700 nm. 

### 4.4. Infrared Absorption Spectra

Infrared absorption spectra of the gels were determined using a Jasco-FTIR 610 FTIR spectrometer, with an attenuated reflection device (ATR), for wet gel samples placed on the ATR window. Due to the high content of water for obtaining absorption spectra for the main compounds of the gels, the samples were dried in an oven at a temperature of 30 °C. The resolution was 2 cm^−1^ in the range of 4000–500 cm^−1^.

### 4.5. SEM Microscopy

The surface morphology of gels was analyzed using the scanning electron microscope SEM (Inspect S, FEICompany), at different magnifications.

### 4.6. Antimicrobial Testing of Bleaching Gels

The microorganisms tested in this study were: Streptococcus mutans ATCC 25175, Porphyromonas gingivalis ATCC 33277, Enterococcus faecalis ATCC 29212, Escherichia coli ATCC 25922 and Staphylococcus aureus ATCC 25923, from the collection of the Laboratory of Microbiology, Faculty of Biology and Geology, UBB, Cluj.

Each bacterial strain was grown for 24 h on a Nutrient Agar medium. A dilution of 0.5 McFarland in sterile saline was then made from each strain. From these dilutions, each Petri dish is inoculated with a sterile swab soaked in the 0.5 McFarland microbial suspension and spread over the entire surface of the solid culture medium (Mueller Hinton-Oxoid), after which the dishes were incubated for 20 min at 37 °C. Then, with a micropipette and sterile cut tips, wells were made in the solid culture medium with a 6 mm diameter each. Subsequently, in each well, the control samples and the samples with the prepared test gels were added with a syringe. Incubation was performed for 24 h at 37 °C. The reading was made by measuring the diameter of the inhibition zone: the larger the diameter of the inhibition zone, the greater the sensitivity of the bacterium to the respective antibacterial substances.

## Figures and Tables

**Table 1:** Diameter of inhibition areas (mm) of the tested gel samples.

Sample	G2	G3	G6	G5
Bacteria	Bromelain Gel	Bromelain Gel	SiO_2_ Gel	Lubrizol Gel
Streptococcus mutans	0	0	0	11
Porphyromonas gingivalis	9	11	0	10
Enterococcus faecalis	8	12	0	11
Escherichia coli	0	9	0	0
Staphylococcus aureus	7	11	0	10

**Table 2:** Chemical composition of the developed experimental gels.

Gels/Composition (g/10 g Gel)	PEG400	SiO_2_	Quince Extract	Bromelain	Whey	Nanoencapsulated Bromelain	Lubrizol	H_2_O
G1	2.216	1.222	7.042	0.2	-	-	-	-
G2	2.216	1.222	-	0.2	0.2	-	-	6
G3	-	-	-	0.2	0.5	-	1	11.5
G4	-	-	-	-	0.2	2	1	14
G5 (Carbopol)	-	-	-	-	-	-	0.25	10
G6 (SiO_2_)		1.22						10
G7 (Bromelaina)				0.2				10
